# Hidden Hazards: Blood Lead and Cadmium Levels Associated with Biochemical and Hematological Markers

**DOI:** 10.34172/apb.025.46563

**Published:** 2026-01-06

**Authors:** Mina Islambulchilar, Ali Shayanfar, Meisam Bagheri, Omid Mehri

**Affiliations:** ^1^Department of Pharmacology and Toxicology, Faculty of Pharmacy, Tabriz University of Medical Sciences, Tabriz, Iran; ^2^Zanjan Applied Pharmacology Research Center, Zanjan University of Medical Sciences, Zanjan, Iran; ^3^Pharmaceutical Analysis Research Center, Tabriz University of Medical Sciences, Tabriz, Iran; ^4^Department of Medicinal Chemistry, Faculty of Pharmacy, Tabriz University of Medical Sciences, Tabriz, Iran; ^5^Student Research Committee, Zanjan University of Medical Sciences, Zanjan, Iran

**Keywords:** Lead, Cadmium, Gas station workers, Male reproductive hormones, Oxidative stress biomarkers

## Abstract

**Introduction:**

Workers occupationally exposed to fuel vapors are at risk of contamination by heavy metals such as lead and cadmium. This study aimed to quantify blood levels of lead and cadmium and evaluate associated biochemical, hormonal, and oxidative stress alterations among fuel station workers.

**Methods:**

This cross-sectional study recruited 66 healthy males, comprising 33 workers with at least 2 years of direct fuel exposure and 33 non-exposed controls. Blood lead and cadmium were quantified using Flame Atomic Absorption Spectrophotometry (AAS). Biochemical markers, reproductive hormones, and oxidative stress indicators were analyzed. Statistical analyses were performed using independent t-tests to identify significant differences between groups, and Pearson’s correlation test to assess associations.

**Results:**

Mean blood lead (43.4±5.4 µg/dL vs. 23.6±1.6 µg/dL; *P*<0.05) and cadmium levels (10.1±1.2 µg/dL vs. 5.5±0.4 µg/L; *P*<0.05) were significantly higher in exposed workers. Biochemical markers were elevated including ALT (38.76±3.3 U/L vs. 24.39±3.6 U/L; *P*<0.05), AST (29.91±3.4 U/L vs. 16.55±4.0 U/L; *P*<0.05), ALP (82.82±12.4 U/L vs. 42.67±9.5 U/L; *P*<0.05), BUN (19.52±3.1 v mg/dL s. 9.82±2.0 mg/dL), and Creatinine (1.13±0.1 mg/dL vs. 0.72±0.1 mg/dL; *P*<0.05) in exposed group. Oxidative stress was evident with higher MDA (2.86±0.6 nmol/mL vs. 0.84±0.2 nmol/mL; *P*<0.05) and lower SOD and GSH (*P*<0.05). Testosterone decreased (3.77±0.9 ng/mL vs. 4.98±0.9 ng/mL; *P*<0.05) while FSH increased (*P*<0.05).

**Conclusion:**

Occupational exposure to lead and cadmium suggests adverse hepatic, renal, hormonal, and oxidative stress changes among fuel station workers. Regular monitoring of biochemical and hormonal markers is strongly recommended, along with preventive exposure-control measures.

## Introduction

 The presence of xenobiotics in the environment poses a serious threat to the physiological integrity of living organisms, since these compounds can penetrate the biological systems through different ways of exposure: inhalation, ingestion, dermal contact, and diffusion.^1^ Among the multitude of xenobiotics, gasoline, a complex petroleum product derived from crude oil, stands out for its composition of volatile and flammable compounds, including aliphatic, halogenated, and aromatic hydrocarbons. Notably, gasoline also contains toxic contaminants such as benzene, toluene, ethylbenzene, and xylene (usually denominated BTEX), and heavy metals like lead and cadmium.^2,3^ The growing industrial activities and continuous use of gasoline have increased human exposure, especially among workers in fuel stations who come into direct contact with petroleum products.^4^

 The health risks of exposure to petroleum distillates, especially those contaminated with metals, are serious and require quick attention. Occupational contact mainly happens via inhalation and skin contact, while urinary excretion and exhalation are the primary routes of elimination.^5^ However, prolonged low-level exposure may lead to bioaccumulation and long-term toxicity.

 Many studies indicate that gasoline exposure negatively affects various organ systems, posing serious health risks for fuel station attendants compared with non-exposed individuals.^6^ Since fuel stations represent critical points of occupational contact, the associated health risks are a growing public health concern. Although BTEX levels at Iranian fuel stations were reported to be within permissible limits,^7^ the cumulative contact with metals remains poorly addressed.

 Heavy metals, particularly lead and cadmium, are widespread environmental pollutants posing serious health hazards in industrial settings. These metals can be absorbed through inhalation or ingestion, causing systemic accumulation, toxicity, and increased morbidity. The World Health Organization (WHO) lists lead among the major public health concerns.^8^ Exposure to lead, whether acute or chronic, is linked to damage in multiple organ systems, including the nervous, reproductive, circulatory, renal, hepatic, and hematopoietic systems.^9^ Similarly, cadmium exposure can disrupt enzymatic pathways, induce oxidative stress, and cause renal and hepatic injury.^9,10^ Recent studies have also demonstrated associations between lead and cadmium exposure and male reproductive dysfunction and increased oxidative stress.^11^

 This study aims to evaluate the risk among fuel station workers due to occupational contact with lead and cadmium via inhalation and ingestion. The objectives are to determine the blood levels of these metals and assess their effects on biochemical and hormonal parameters, particularly male reproductive hormones and oxidative stress biomarkers. This investigation is important because fuel station workers are continuously exposed to hazardous substances that may compromise their health and quality of life. Understanding exposure levels and biological effects will guide the development of effective occupational health and safety strategies.

 As industrialization accelerates, the number of individuals exposed to toxic agents in workplaces is increasing. The findings of this study are expected to provide valuable data for regulatory authorities and health policymakers to strengthen workplace safety standards. Additionally, these results may serve as a foundation for public health interventions aimed at reducing contact and protecting vulnerable populations.^8,12^

 This research adopts a comprehensive approach to evaluate the specific health risks associated with lead and cadmium exposure among fuel station workers, a group that remains underrepresented in scientific literature. Although previous studies have discussed the general health effects of gasoline exposure, few have specifically examined the combined influence of metals on oxidative stress and male reproductive health. By integrating these parameters, this study seeks to clarify the biological mechanisms underlying heavy metal toxicity and to provide evidence supporting improved occupational monitoring and regulatory control in the petroleum industry.^10,11^

## Materials and Methods

###  Study Population

 This is a cross-sectional case-control study conducted in Zanjan, Zanjan, Iran, and received ethical approval from the local Research Ethics Committee of Zanjan University of Medical Sciences & Institutional Review Board (IR.ZUMS.REC.1397.361 & IR.ZUMS.REC.1397.357). The principles stated in the Declaration of Helsinki were considered in this study. Written informed consent was taken from all participants before their recruitment.

 The study population included male workers occupationally exposed to fuel vapours at four operational fuel stations. A total of 60 workers were initially approached. After eligibility screening, 45 met the inclusion criteria; however, 12 individuals declined to participate due to personal or work-related reasons, resulting in 33 participants who consented and were enrolled. Inclusion criteria were healthy males aged 20 to 50 years, with no underlying medical conditions, medications, supplements, or use of substances, including alcohol or other addictive substances. Participants had to have at least two years of direct occupational contact to fuel and work for a minimum of 8 hours per day. The non-exposed group consisted of 33 healthy males of comparable age working at least 8 hours per day in grocery stores, with no direct exposure to fuel or fuel vapours. Thus, the final study population included 66 participants (33 exposed, 33 non-exposed). All participants were examined by a urologist to exclude any underlying reproductive disorders.

###  Study Variables

 Personal data were collected from all participants, including age, smoking status and duration, job experience, previous employment, marital status, and socioeconomic status.

####  Chemicals and Sample Containers

 Analytical-grade reagents, including nitric acid (37%) and hydrogen peroxide (30%), were obtained from Merck, Germany. Stock standard solutions for cadmium and lead were prepared using licensed standard solutions (1000 ppm, Merck, Germany).

 All glassware and polyethylene plastic containers used were cleaned and then soaked in 10% (v/v) nitric acid for 24 h, followed by thorough rinsing with deionized water and drying in an oven before use.

####  Hematological Parameters

 Venous blood samples (10 mL) were collected under aseptic conditions. One milliliter was transferred into EDTA tubes to analyze hematological parameters (red blood cells, white blood cells, hemoglobin, hematocrit, and mean corpuscular volume) using a Sysmex® Automated Hematology Analyzer (Sysmex Co., Japan). Anemia was defined as a hemoglobin level below 13 g/dL.

 The remaining 9 mL of blood serum were then centrifuged at 3000 rpm for 5 min, frozen and stored at -20ºC, and subsequently used in the biochemical assay, including liver and kidney function tests, reproductive hormones, oxidative stress biomarkers, and measurement of lead and cadmium concentration.

####  Oxidative Stress Markers

 Malondialdehyde (MDA) levels were measured by a modified thiobarbituric acid (TBA) test, as described by Tualeka et al.^13^ Superoxide dismutase (SOD) and total antioxidant capacity (TAC) levels were measured using enzyme kits from Biorexfars, Iran.^13^

####  Reproductive Hormones

 Serum concentrations of reproductive hormones, in particular follicle-stimulating hormone (FSH), luteinizing hormone (LH), and testosterone, were quantitatively measured using the IMMULITE 2000 Immunoassay System (Siemens, Germany). Serum samples were thawed and mixed well to ensure homogeneity before analysis. The immunoassay was performed per the manufacturer’s instructions and included the preparation of calibration standards and quality control samples to ensure the accuracy and reliability of the assay. The detection limits for FSH, LH, and testosterone were established, and all samples were run in duplicate to keep variability low. The results were expressed in international units per liter (IU/L) for FSH and LH, and in nanograms per milliliter (ng/mL) for testosterone. The intra-assay and inter-assay coefficients of variation were kept under control, which confirmed the precision of the measurements made.

####  Biochemical Assays

 Serum liver function tests (LFTs), including bilirubin, serum glutamate oxaloacetate transaminase (SGOT), and serum glutamate pyruvate transaminase (SGPT), as well as kidney function tests (KFTs), including urea and creatinine, were measured using the Roche Hitachi 917 Multichannel Analyzer (Hitachi, Japan) with reagents supplied by Roche Diagnostics (Mannheim, Germany). Two millilitres of blood were digested using the conventional wet acid digestion method (CDM) as described by Memon et al.^14^

####  Metal Levels

 Blood lead and cadmium concentrations were determined by flame atomic absorption spectrometry (Chemtech AA Spectrophotometer, model CTA 3000) at wavelengths 283.3 nm (lead) and 228.8 nm (cadmium), with atomization temperatures set at 2000ºC and 1550ºC, respectively. Calibration curves were prepared using diluted certified standards (1–10 µg/L for Cd; 10–60 µg/L for Pb).

###  Statistical Analysis

 Data were analysed using SPSS version 26 (IBM Corp., Armonk, NY, USA). Descriptive statistics (mean ± SD, frequencies) were calculated. Chi-square and independent t-tests assessed group differences; Mann–Whitney U tests were used when normality assumptions were violated. Significance was set at *P* < 0.05 (two-tailed).

## Results

**Figure 1:**
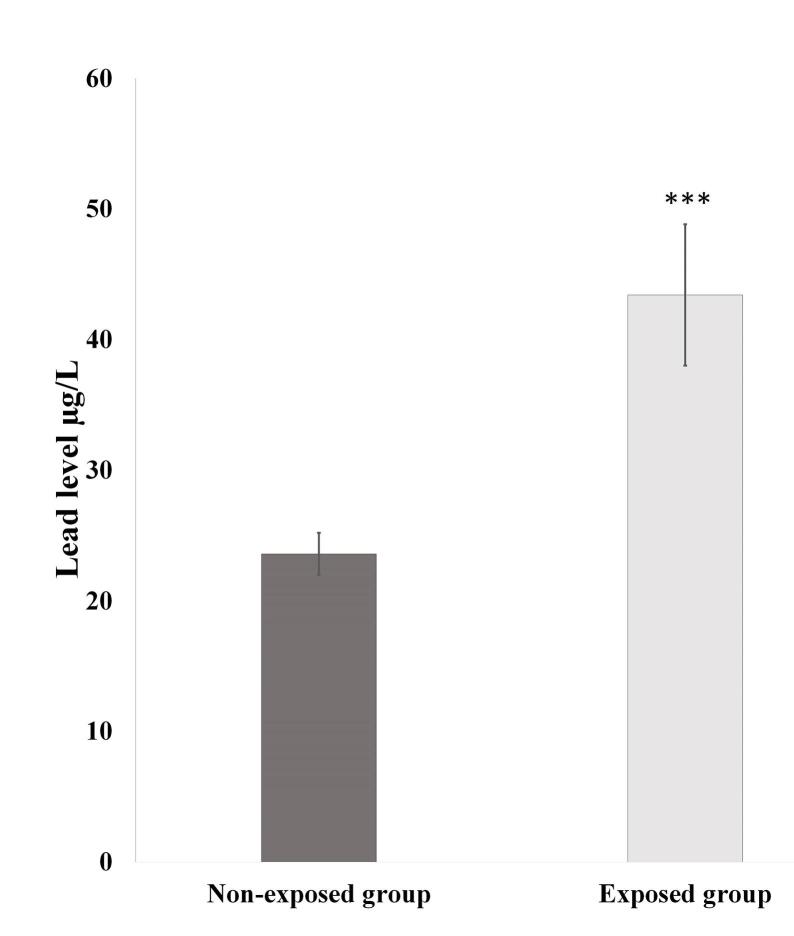
Blood lead levels in both study groups. *P* values lower than 0.05 are considered significant

**Figure 2:**
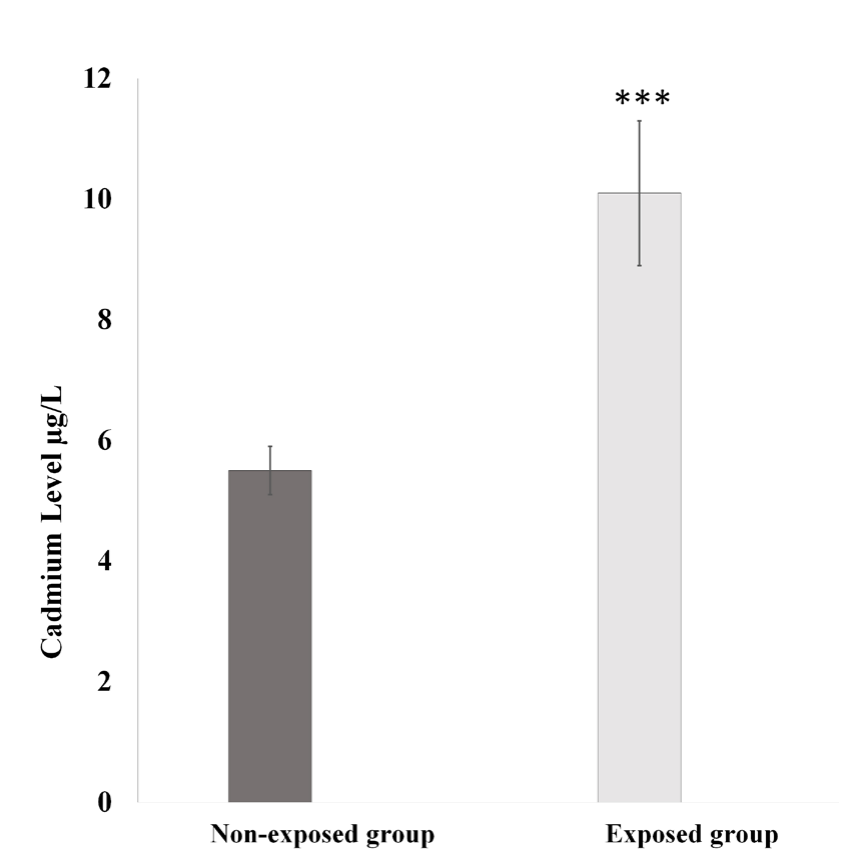
Blood cadmium levels in both study groups. *P* values lower than 0.05 are considered significant

 In this study, we aimed to assess the health impact of exposure to petroleum products on male workers by comparing an exposed group (fuel station workers) with a non-exposed group (grocery store workers). Of a total of 60 male fuel station workers who had direct contact with petroleum products, 45 met the inclusion criteria, and 33 agreed to participate as the exposed group. The non-exposed group consisted of 33 healthy males employed in grocery stores without direct fuel contact. The two groups were matched for age and other demographic characteristics. The demographic characteristics of both groups were summarized as follows: the mean age of the exposed group was 28.58 ± 6.50 years, while the mean age of the non-exposed group was 28.33 ± 5.73 years, with no statistically significant difference (*P* > 0.05). Similarly, mean work experience did not differ significantly between the exposed group (5.9 ± 3.8 years) and the non-exposed group (6.2 ± 4.9 years). Regarding smoking habits, 39% of the exposed group were smokers (3% heavy smokers, 6% one pack per day, and 30% fewer than 10 cigarettes per day), compared with 21% in the non-exposed group (3% heavy smokers, 6% one pack per day, and 12% fewer than 10 cigarettes per day). Marital status was also similar between groups, with 61% of the exposed group and 67% of the non-exposed group being married. The biochemical analyses showed that blood lead and cadmium levels were significantly higher in the exposed group compared to the non-exposed group (*P* < 0.05, both; see Figures 1 and 2). Moreover, the levels of oxidative stress markers were assessed (see Table 1), and it was indicated that the malondialdehyde (MDA) level was significantly higher in the exposed group (*P* = 0.00). On the other side, superoxide dismutase (SOD) and glutathione (GSH) levels were significantly higher in the non-exposed group (*P* < 0.05 for both). Remarkably, there was a positive correlation of MDA levels with both lead and cadmium concentrations (*P* < 0.05 for both), while the levels of SOD and GSH were negatively correlated with lead and cadmium levels (*P* < 0.05, both).

**Table 1:** Oxidative stress biomarkers in both study groups.

**Parameter**	**Exposed group** **Mean±SD**	**Non-exposed group ** **Mean±SD**
MDA (nmol/mL)	2.86 ± 0.6***	0.84 ± 0.2
SOD (U/mL)	8.70 ± 2	11.97 ± 1.4***
GSH (mg/dl)	21.4 ± 3.1	32.70 ± 3.2***

*P* values lower than 0.05 are considered significant. *Different (higher) Significantly at *P* < 0.05. **Different (higher) Significantly at *P* < 0.01. ***Different (higher) Significantly at *P* < 0.001.

 The hematological parameters and biochemical assays for both groups are presented in Table 2. A negative correlation was identified between erythrocyte and platelet counts and blood lead (*P* < 0.05, both) and cadmium levels (*P* < 0.05 for both). Similarly, the hemoglobin (Hb) and hematocrit (Hct) levels were negatively correlated with blood lead (*P* < 0.05, both) and cadmium levels (*P* < 0.05, both). In contrast, leukocyte counts were positively associated with blood lead and cadmium levels (*P* < 0.05 for each).

**Table 2:** Hematological and biochemical parameters in both study groups.

**Parameter**	**Exposed group** **Mean±SD**	**Non-exposed group ** **Mean±SD**
RBC ( × 10^12^/L)	4.7 ± 0.1	5.4 ± 0.2***
WBC ( × 10^9^/L)	7.5 ± 0.2***	6.5 ± 0.2
PLT ( × 10^9^/L)	219.8 ± 26.8	333.9 ± 36.2***
Hb (g/dL)	13.4 ± 0.3	15.3 ± 0.5**
Hct (%)	37.4 ± 3.1	45.8 ± 1.9***
Bil (mg/dL)	0.85 ± 0.1***	0.53 ± 0.1
ALT (U/L)	38.76 ± 3.3***	24.39 ± 3.6
AST (U/L)	29.91 ± 3.4***	16.55 ± 4.0
ALP (U/L)	82.82 ± 12.4***	42.67 ± 9.5
BUN (mg/dL)	19.52 ± 3.1***	9.82 ± 2.0
Creatinine (mg/dL)	1.13 ± 0.1***	0.72 ± 0.1

*P* values lower than 0.05 are considered significant. *Different (higher) Significantly at *P* < 0.05. **Different (higher) Significantly at *P* < 0.01. ***Different (higher) Significantly at *P* < 0.001.

 Further analysis showed a negative relation of erythrocyte and platelet counts, Hb, Hct, and MDA levels (*P* < 0.05 for all). On the other hand, erythrocyte and platelet counts, Hb, and Hct levels showed positive correlations with SOD (*P* < 0.05 for all) and GSH (*P* < 0.05for all).

 Liver function tests, including alanine aminotransferase (ALT), aspartate aminotransferase (AST), alkaline phosphatase (ALP), and bilirubin, showed positive correlations with blood lead (*P* < 0.05 for all) and cadmium levels (*P* < 0.05 for all). They were also respectively negatively correlated with SOD (*P* < 0.0001 for all) and GSH levels (*P* < 0.0001 for all), whereas they showed positive correlation with MDA levels (*P* < 0.0001 for each).

 Furthermore, there was an inverse correlation of BUN and creatinine levels with SOD (*P* < 0.0001 for both) and GSH levels (*P* < 0.0001 for both), and a positive correlation with MDA levels (*P* < 0.0001 for both).

 The male reproductive hormone levels for both study groups are presented in Table 3. There was also a significant negative correlation of blood lead levels with testosterone (*P* < 0.05). Besides, the levels of follicle-stimulating hormone (FSH) showed significant positive correlations with blood lead and cadmium levels (*P* < 0.0001 for both). No statistically significant correlation was found between luteinizing hormone (LH) levels and either metal levels or oxidative stress markers.

**Table 3:** Male reproductive hormones in both study groups.

**Parameter**	**Exposed group** **Mean±SD**	**Non-exposed group ** **Mean±SD**
Testosterone (ng/mL)	3.77 ± 0.9	4.98 ± 0.9***
FSH (IU/L)	4.27 ± 0.9***	2.98 ± 0.8
LH (IU/L)	4.7 ± 1.7	4.8 ± 1.7

*P* values lower than 0.05 are considered significant. *Different (higher) Significantly at *P* < 0.05. **Different (higher) Significantly at *P* < 0.01. ***Different (higher) Significantly at *P* < 0.001.

 MDA levels were negatively correlated with testosterone (*P* < 0.0001) and positively correlated with FSH levels (*P* < 0.0001). No significant correlations were found between LH levels, metal concentrations, and oxidative stress markers.

## Discussion

 To our knowledge, this study is the first to investigate blood lead and cadmium levels among petroleum-exposed workers at fuel stations in Iran, focusing on their non-carcinogenic effects on various body systems. Our findings indicate that the exposed group has significantly higher blood lead and cadmium levels than the non-exposed group. This elevation is concerning because both metals are well known to induce oxidative stress, which, in our study, showed a significant correlation with lead and cadmium concentrations. Likewise, the exposed group exhibited lower testosterone and higher follicle-stimulating hormone (FSH), both of which were significantly correlated with blood lead, cadmium, and oxidative stress biomarkers.

 These findings align with previous reports highlighting the occupational hazards of metals, particularly lead and cadmium.^15-17^ Despite the use of unleaded fuel in many regions, residual contamination persists, and the reduction of body lead burden remains a slow process.^18^ The observed biochemical and hormonal changes reinforce the evidence that chronic occupational contact with heavy metals contributes to oxidative stress, endocrine disruption, and other systemic toxicities.^19-22^

###  Hematological Effects

 Our study substantiates the previous report on the hematotoxic effects of occupational exposure to petroleum products. We found a statistically significant decrease in the red blood cell (RBC) count, hemoglobin (Hb), hematocrit (Hct), and platelet counts among the exposed group compared with the non-exposed group. Conversely, although the total white blood cell (WBC) count was higher in the non-exposed group, this difference was not statistically significant. Similar studies have been conducted in Egypt,^23^ Ethiopia,^24^ Solymanie (Iraq),^25^ and Brazil,^26^ have reported significant alteration in hematological parameters following occupational contact, while studies from Tie-Qar (Iraq),^27^ and Kerman (Iran),^28^ demonstrated increased hematological parameters in exposed workers. These inconsistencies may relate to variations in the lead content of local fuels, exposure duration, and environmental factors. For example, a study from Shiraz that found no significant differences in Hb concentrations after a minimum of one year of contact indicates that exposure duration and fuel composition are crucial determinants of hematological outcomes.^29^

 Another study demonstrated a relationship between decreased hematological indices and exposure durations of less than two years.^24^ The reduction in RBC count and Hb levels may be attributed to shortened erythrocyte survival and disrupted heme synthesis.^30^ These hematological variations may also reflect differing levels of oxidative stress and exposure to metals, particularly lead, and to hydrocarbons such as benzene and its metabolites.^31^ Reactive oxygen species (ROS) and metabolic byproducts of benzene and other hydrocarbons can damage the RBC membrane, leading to reduced cell size and impaired protein synthesis, including heme production in the bone marrow.^2^

###  Hepatic and Renal Implications

 Similarly, our results revealed significantly elevated levels of total and direct bilirubin, alanine transaminase (ALT), aspartate transaminase (AST), and alkaline phosphatase (ALP) in the exposed group, which were significantly correlated with serum lead and cadmium levels and oxidative stress biomarkers. These findings are consistent with previous studies conducted in Ethiopia,^32^ Egypt,^23,33^ Iraq,^34^ and Sudan,^35^ where similar liver function abnormalities were observed following metal exposure.

 The hepatotoxic effects of lead and cadmium are well-documented.^2,19^ Both metals accumulate in the liver, where they impair hepatocyte membrane integrity, induce mitochondrial dysfunction, disrupt calcium homeostasis, and generate reactive oxygen species (ROS), leading to promoted lipid peroxidation and disrupted antioxidant defenses, protein oxidation, and DNA damage in hepatocytes.^6,19-21,32,36-42^ Cadmium, in particular, depletes glutathione (GSH) and binds to sulfhydryl groups, inactivating antioxidant enzymes such as superoxide dismutase (SOD) and catalase, thereby compromising cellular defense systems.^42^ Lead interferes with heme synthesis and impairs energy production by inhibiting key enzymes like δ-aminolevulinic acid dehydratase (ALAD) and cytochrome oxidase. The observed increases in ALT and AST suggest hepatocellular injury, while elevated ALP may reflect impaired biliary excretion or increased osteoblastic activity secondary to bone turnover, a known site of lead deposition.^36,38^ Together, these enzyme elevations indicate early-stage hepatic impairment associated with chronic low-level contact.

 In our study, the exposed group exhibited significantly elevated levels of blood urea and creatinine compared with the non-exposed group, suggesting early renal stress associated with heavy metal exposure. These parameters also showed positive correlations with blood lead and cadmium levels as well as MDA, alongside inverse correlations with antioxidant markers (SOD and GSH). Such patterns indicate oxidative stress-mediated renal impairment. This is biologically plausible given the well-documented nephrotoxicity of lead and cadmium, which accumulate in renal tissue over time, particularly within the proximal convoluted tubules.^19^ Once deposited, these metals induce mitochondrial dysfunction, generate reactive oxygen species (ROS), and disrupt antioxidant defence systems, ultimately resulting in lipid peroxidation, tubular necrosis, and impaired glomerular filtration. Previous studies have similarly reported increases in BUN and creatinine among occupationally exposed workers, supporting the likelihood of nephron damage and reduced GFR under chronic contact conditions.^23,43-45^ Elevated urea and creatinine in our study may indicate nephrotoxicity associated with chronic exposure, consistent with other reports describing renal impairment in workers exposed to metals.^23,46^ Lead and cadmium have a high affinity for sulfhydryl groups, enabling them to interfere with enzymatic processes and structural proteins in renal epithelial cells, which may explain the observed biochemical alterations.^45^ Reduced levels of antioxidant enzymes observed in our exposed group further emphasize the role of oxidative injury in renal dysfunction progression.

 Collectively, these findings underscore that even low-to-moderate chronic contact may contribute to subclinical nephrotoxicity with potential long-term consequences, including an increased risk of chronic kidney disease. Therefore, routine monitoring of renal biomarkers, implementation of occupational safety measures, and minimizing metal exposure are warranted to protect vulnerable workers.

###  Endocrine Disruption

 Hormonal analysis revealed that the exposed group had lower testosterone and higher FSH levels, both of which were significantly correlated with oxidative stress biomarkers and metal concentrations. These hormonal imbalances may result from the combined toxic effects of BTEX compounds and metals, which are known to induce oxidative stress and testicular damage.^22,37^ Heavy metal exposure can disrupt the hypothalamic–pituitary–gonadal axis, altering hormone regulation and contributing to reproductive dysfunction.^47^ Prior studies support these findings, reporting similar endocrine disruptions among occupationally exposed populations. For example, Saadat and Monzavi and Saadat et al. observed decreased testosterone levels in workers occupationally exposed to heavy metals.^48,49^ Conversely, Jouda et al. and Dohi & Abdulhay emphasized the association between metal contact and altered reproductive hormone levels.^50,51^

###  Oxidative Stress Markers

 Our evaluation of oxidative stress biomarkers demonstrated that the malondialdehyde (MDA) levels were significantly elevated, while the superoxide dismutase (SOD) and glutathione (GSH) levels were significantly reduced in the exposed group. These results suggest that chronic exposure to petrol vapors induces oxidative stress, a condition where the balance between reactive oxygen species (ROS) production and antioxidant defences is disrupted.^52,53^ Increased MDA levels, a marker of lipid peroxidation, indicate enhanced oxidative damage to cellular membranes, whereas reduced SOD and GSH activities reflect a compromised antioxidant capacity.^54^ Similar findings of increased oxidative stress markers among individuals exposed to these metals and organic solvents have been reported in several studies.^39,55^

###  Metal Levels

 Blood lead and cadmium levels were significantly higher in the exposed group, likely due to higher ambient concentrations of these metals in the workplace.^56^ Our results also revealed a positive correlation between the employment duration and blood metal concentrations, indicating that longer contact periods increase the risk of metal accumulation in the body. This finding aligns with previous studies in Nigeria, reporting similar trends.^57^ Conversely, Alrumaidh and Enayah found no significant association between duration of employment and heavy metal levels,^34^ suggesting that individual susceptibility and environmental conditions also influence metal accumulation. These findings highlight the need for continued research to identify key determinants of metal bioaccumulation in occupational settings.

## Limitations

 This study has certain limitations that should be acknowledged. First, its cross-sectional design limits our ability to establish causal relationships between metal exposure and observed biochemical or hormonal alterations. Second, the sample size, although sufficient to detect significant differences in primary outcomes, is relatively small and may limit the statistical power to detect subtle differences and reduce the generalizability to broader populations. Third, although major confounders such as age, smoking status, and the absence of chronic disease were controlled, several unmeasured factors (including dietary habits, physical activity, non-occupational metal, contact, and genetic polymorphisms influencing metal metabolism) were not assessed and may have influenced the observed outcomes. Additionally, individual contact levels were not quantified through environmental air monitoring or personal dosimeters; thus, exposure assessment relied on job title and duration rather than direct environmental measurements of workplace lead and cadmium levels. Future longitudinal studies incorporating environmental monitoring, personal exposure assessment, and larger sample sizes are warranted to confirm these findings and better elucidate long-term health risks.

## Conclusion

 In conclusion, this study suggests significant associations between occupational contact with lead and cadmium and adverse biochemical, hormonal, and oxidative stress outcomes among fuel station workers in Iran.

 Given the observed biochemical changes, regular monitoring of liver and kidney function, periodic metal screening, and workplace exposure-reduction strategies are recommended for early detection and prevention of long-term health consequences in this high-risk occupational group.

 Future longitudinal studies are warranted to clarify causal mechanisms and assess chronic effects and long-term health outcomes, recovery potential, and protective interventions. Strengthening public health policies to minimize occupational and environmental contact remains essential to protect worker and community health.

## Competing Interests

 There is nothing to declare.

## Consent to Publish

 Not applicable.

## Data Availability Statement

 The datasets generated and analyzed during the current study are available from the corresponding author on reasonable request.

## Declaration of Generative AI and AI-Assisted Technologies in the Writing Process

 During the preparation of this work, the authors used Al for assistance with language editing, improving grammar, and refining clarity according to journal style. After using this tool, the authors reviewed and edited the content as needed and took full responsibility for the content of the manuscript.

## Ethical Approval

 The study was reviewed and approved by the Research Ethics Committee of Zanjan University of Medical Sciencesو Institutional Review Board (IR.ZUMS.REC.1397.361 & IR.ZUMS.REC.1397.357) and was performed under the Declaration of Helsinki.
